# Comparison of Acceleration Techniques for Selected Low-Level Bioinformatics Operations

**DOI:** 10.3389/fgene.2016.00005

**Published:** 2016-02-10

**Authors:** Daniel Langenkämper, Tobias Jakobi, Dustin Feld, Lukas Jelonek, Alexander Goesmann, Tim W. Nattkemper

**Affiliations:** ^1^Biodata Mining Group, Faculty of Technology, Bielefeld UniversityBielefeld, Germany; ^2^Sektion für Bioinformatik und Systemkardiologie, Universitätsklinikum HeidelbergHeidelberg, Germany; ^3^Fraunhofer SCAISankt Augustin, Germany; ^4^Bioinformatik und Systembiologie, Justus Liebig UniversityGießen, Germany

**Keywords:** GPU, FPGA, multi-core, parallelization, automatic, high throughput, bioinformatics, sequence analysis

## Abstract

Within the recent years clock rates of modern processors stagnated while the demand for computing power continued to grow. This applied particularly for the fields of life sciences and bioinformatics, where new technologies keep on creating rapidly growing piles of raw data with increasing speed. The number of cores per processor increased in an attempt to compensate for slight increments of clock rates. This technological shift demands changes in software development, especially in the field of high performance computing where parallelization techniques are gaining in importance due to the pressing issue of large sized datasets generated by e.g., modern genomics. This paper presents an overview of state-of-the-art manual and automatic acceleration techniques and lists some applications employing these in different areas of sequence informatics. Furthermore, we provide examples for automatic acceleration of two use cases to show typical problems and gains of transforming a serial application to a parallel one. The paper should aid the reader in deciding for a certain techniques for the problem at hand. We compare four different state-of-the-art automatic acceleration approaches (OpenMP, PluTo-SICA, PPCG, and OpenACC). Their performance as well as their applicability for selected use cases is discussed. While optimizations targeting the CPU worked better in the complex *k*-mer use case, optimizers for Graphics Processing Units (GPUs) performed better in the matrix multiplication example. But performance is only superior at a certain problem size due to data migration overhead. We show that automatic code parallelization is feasible with current compiler software and yields significant increases in execution speed. Automatic optimizers for CPU are mature and usually no additional manual adjustment is required. In contrast, some automatic parallelizers targeting GPUs still lack maturity and are limited to simple statements and structures.

## 1. Introduction

Due to technological developments, like for instance next generation sequencing or advanced lab robotics, in the last 5–10 years, the data volume, recorded from life science experiments has reached new dimensions. Thus, the bioinformatics has to keep its focus not only on algorithmic aspects of bio-data analysis but also on finding new ways to process huge data collections or the community faces the danger that large amounts of data could be wasted (Ro and Re, 2010).

In recent years the steady race for higher clock rates of modern processors slowed down noticeably owed to physical limits for the miniaturization of integrated circuits (Bendavid, 2006). These limitations eventually gave birth to modern multi-core processors that include several processor cores in one processor package. In the following the term *processor* refers to single core CPUs as well as a single core in a multi-core CPU. The challenges faced in hardware design also found their way in software development where an increasing number of applications were adapted for use on computers featuring multiple processors. The very basic idea behind these parallelization techniques is to distribute computing operations to several processors instead of using just one single processor, reducing the running time of an application significantly without the need for higher clock rates. However, this shift of paradigm requires fundamental changes in software design and problem solving strategies in general. In order to achieve reasonable performance when using more than one processor, the algorithm of interest should be described in such a way that as many as possible computations can be processed in arbitrary order. This requirement ensures that data can be processed in parallel instead of classical serial computations, where data is processed in a strict order. Nowadays there are four major techniques concerning optimization and parallelization of applications, namely CPU-multi-processing, Vector instructions and cache optimization, Cluster Computing (Message Passing, job schedulers) and the use of specialized acceleration devices e.g., FPGAs, GPUs, MICs. For most of these strategies manual, automatic, or hybrid parallelization techniques are available.

In the following we present acceleration techniques along with a schematic, showing how acceleration could be realized for the *k*-mer counting problem (Please note that this is only one possible solution for acceleration and a multitude of solutions exist). A *k*-mer is a word of length *k* on a given alphabet, e.g., the DNA alphabet Σ = {*A, C, G, T*}. To extract all possible *k*-mers of a source string, a sliding window of size *k* is moved through the string counting the occurrences. The task of the example employed in this section is to count the occurrences of all 256 4-mers on a given sequence. *k*-mers are the building blocks employed in a multitude of bioinformatics tasks, e.g., phylogenetics (Martin et al., 2008; Diaz et al., 2009), because they provide a compressed representation of a sequence.

## 2. Materials and methods

### 2.1. Techniques

#### 2.1.1. CPU-multi-processing

Multi-core/processor machines are computer systems with more than one processor. They are typically used as workstations or servers. Common desktop computer systems today employ up to eight processors, with servers or workstation using even more. For CPU-multi-processing, two libraries for use with popular programming languages including C, C++, or Fortran dominate software development, namely Posix Threads (pthreads; Butenhof, 1997) and OpenMP (Dagum and Menon, 1998). Both approaches are well established, stable and have an active community. Although both libraries facilitate a parallel execution of code, usage scenarios are slightly different. OpenMP is commonly used by annotating the application's source code with hints on where to optimize, in order to perform an automatic parallelization of suitable language constructs such as loops without any further action of the developer. The pthreads library offers no automatic parallelization. Thread creation/destruction has to be done explicitly thus modifying the code is necessary. OpenMP is often preferred due to the possibility of constructing parallel versions of existing applications, without changes in the application's logic and thus manageable time and effort preserving compatibility for systems without OpenMP due to its directive-based nature.

Special kinds of multi-core hardware are many-integrated-core (MIC) devices. These incorporate many cores on one chip. The advantage is that MICs can be programmed using OpenMP for example.

**Figure 1:**
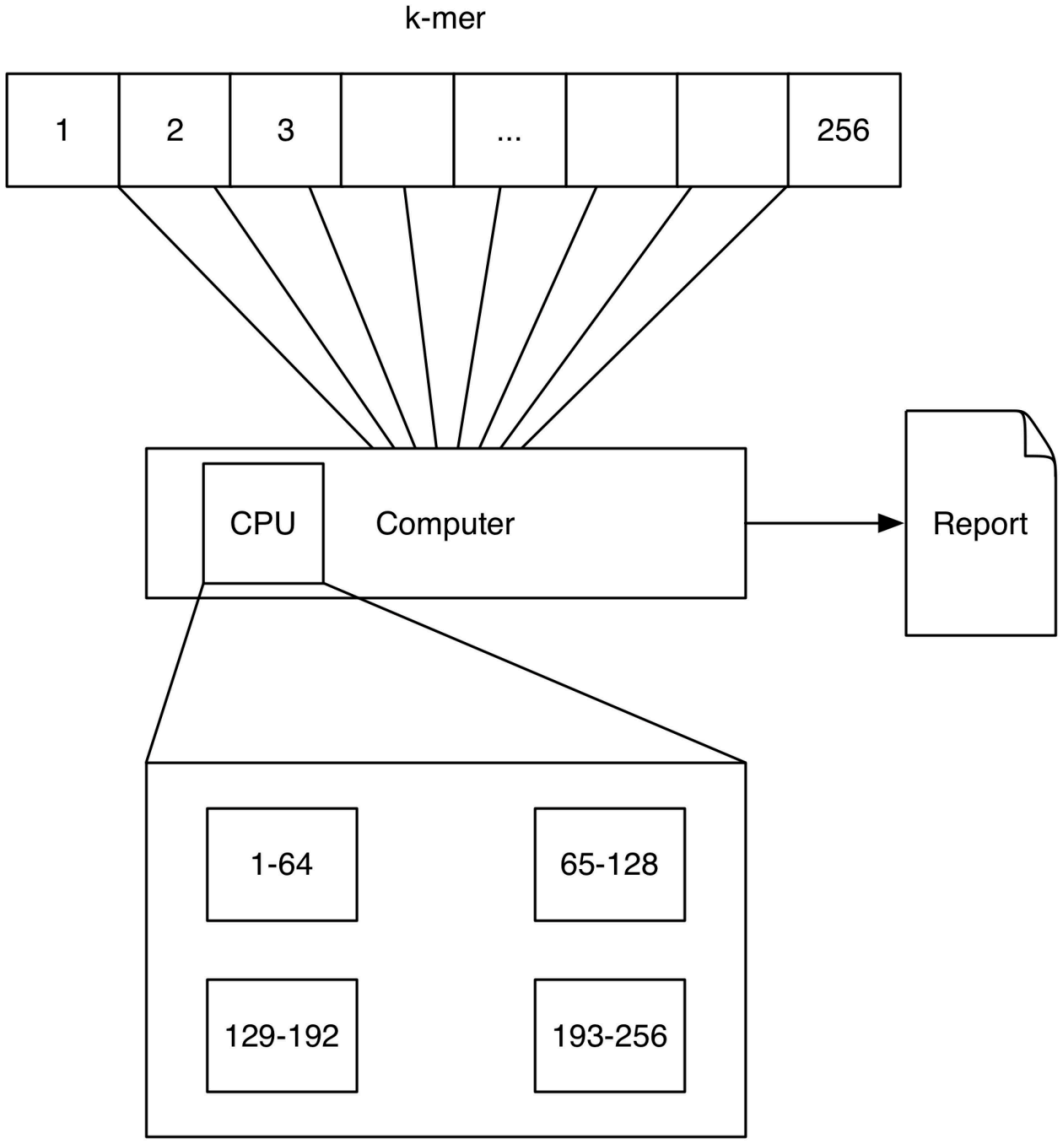
**Multithreading: The 4^***k***^ = 256 4-mers (depicted by the numbers 1–256) are processed on a single computer with four processors (depicted by the rectangular boxes at the bottom)**. Each processor computes a quarter of all *k*-mer counts.

A schematic depicting one possibility of computing *k*-mers using multi-processing is shown in Figure 1. Here each processor computes a quarter of all *k*-mer counts.

#### 2.1.2. Vector instructions and cache optimizations

Recent processors feature several specialized vector instruction sets (e.g., MMX, SSE, AVX) that allow efficient processing of vector data structures often used in visual operations or in scientific computing. These instruction sets employ data parallelism to enable the execution of operations on the vector structure at once instead of performing the operation on each element of the vector subsequently, which results in a performance gain for typical vector and matrix operations, e.g., dot products. Unfortunately, the quality of transformation of applications to use vector instructions is highly dependent on the compiler used and the code complexity.

Nowadays the size of memory in server systems is sufficient for a multitude of algorithmic problems. However, access time and bandwidth of main memory may introduce bottlenecks. Placed as intermediate memory between main memory and processor, the processor's cache acts as a fast buffer for reoccurring memory accesses and avoids CPU access to the slower main memory. The transfer of memory contents is performed in chunks, so called cache lines. The caching mechanism works well if the access to the information in the cache is continuous but fails in case of random access to the memory.

Cache optimizers attempt to avoid unnecessary data transfers by optimizing accesses to the memory to be continuous. Manual optimization requires substantial development time, knowledge, experience, and resources and may therefore be left to suitable software, such as PluTo-SICA.

PluTo-SICA (Feld et al., 2013, 2015) transforms annotated (similar to OpenMP) source code to utilize vector instructions as well as perform cache optimizations and additionally can parallelize the application using OpenMP (see CPU-multi-processing).

**Figure 2:**
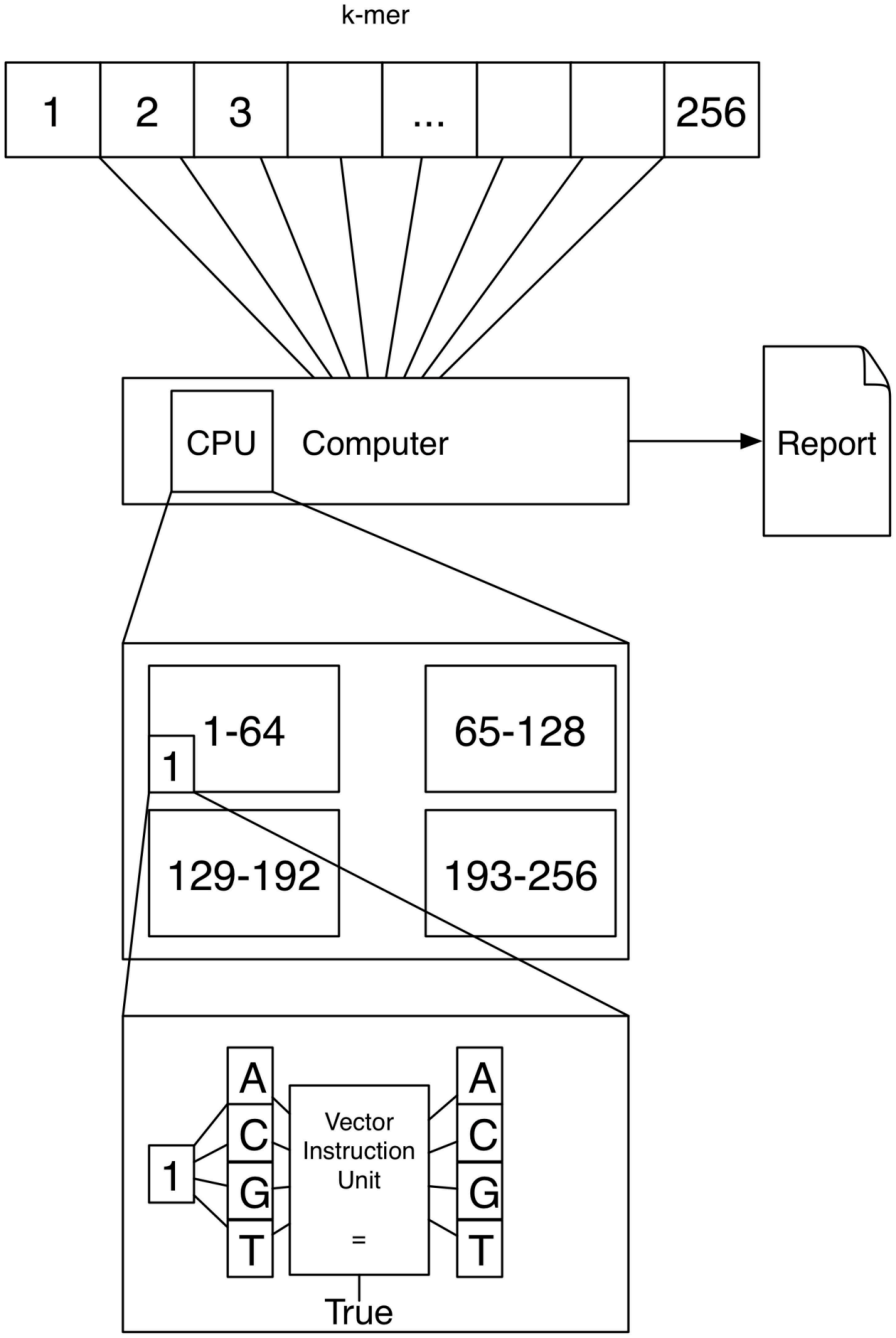
**Vector instruction units are located inside a processor and can execute a single instruction on multiple data at once**. This means that for example comparing four character-pairs is (almost) as fast with vector instructions as comparing 1 character-pair.

A schematic depicting one possibility of computing *k*-mers using vector instructions is shown in Figure 2. If *k* = 4 a vector instruction could compare all four characters of the 4-mer to 4 characters of the text instead of using a for loop comparing one character-pair at a time.

#### 2.1.3. GPUs

Nowadays, GPUs capable of being used for scientific computations [General Purpose GPU (GPGPU) computing] become more and more prevalent in research workstations. They are different from CPUs as they are specifically designed for highly parallel computations and possess a much higher number of processors than CPUs (e.g., NVIDIA Tesla K40: 2880 processors; NVIDIA Corporation, 2014) and generally provide a higher bandwidth to the memory.

Although GPUs feature a vast number of processors and have a high memory bandwidth, not all algorithms can be efficiently run on GPUs. Algorithms have to be SIMT conformant and random global memory access must be coalesced in order to be efficient. Furthermore, latency hiding of memory access might be an issue, which is compensated for a bit on modern GPUs by utilizing cache architectures (cmp. NVIDIA, 2015). Moreover, deep nested control structures are inefficient. Applications requiring double precision for floating point numbers will have significant performance penalty depending on the GPU utilized.

Two APIs, CUDA (NVIDIA Corporation, 2013) and OpenCL (Khronos OpenCL Working Group, 2014), established their claim in GPU programming. CUDA is more established and offered the best performance in the past, but is limited to NVIDIA GPUs, whereas OpenCL is compatible to a wider range of hardware (NVDIA/ATI GPUs as well as other devices, e.g., CPUs, MICs) and continuously gains ground in terms of performance (Karimi et al., 2010; Fang et al., 2011). In Komatsu et al. (2010) diagnosed that the performance difference is due to missing compiler optimizations in the OpenCL C Compiler. A recent benchmark of the SHOC benchmark suite (Danalis et al., 2010) shows that in the MD5 hash benchmark the performance of OpenCL and CUDA is comparable, but CUDA is significantly faster in the FFT Benchmark (OpenBenchmarking.org, 2015).

Producing GPU accelerated source code requires a great extend of experience and knowledge in GPU hardware design to unlock the full potential of GPUs.

Optimizers specifically tailored to generate GPU code are still relatively unestablished. However, two examples of automatic code generators, PPCG (polyhedral parallel code generation; Verdoolaege et al., 2013) and OpenACC (Open Accelerators; OpenACC Consortium, 2013) exist. PPCG analyzes the source code of an auspicious code fragment and generates the corresponding accelerator code, but is limited in supporting only a subset of programming language constructs. OpenACC is an annotation-based library, loosely resembling OpenMP that automatically generates accelerated code for GPUs. Until now compiler support for OpenACC is limited to a few commercially available compilers such as the Cray Compiling Environment (CCE, Cray Inc, USA), the PGI Accelerator (NVIDIA Corporation, USA, limited university developer license is available free of charge) and the CAPS compiler (CAPS entreprise, France).

**Figure 3:**
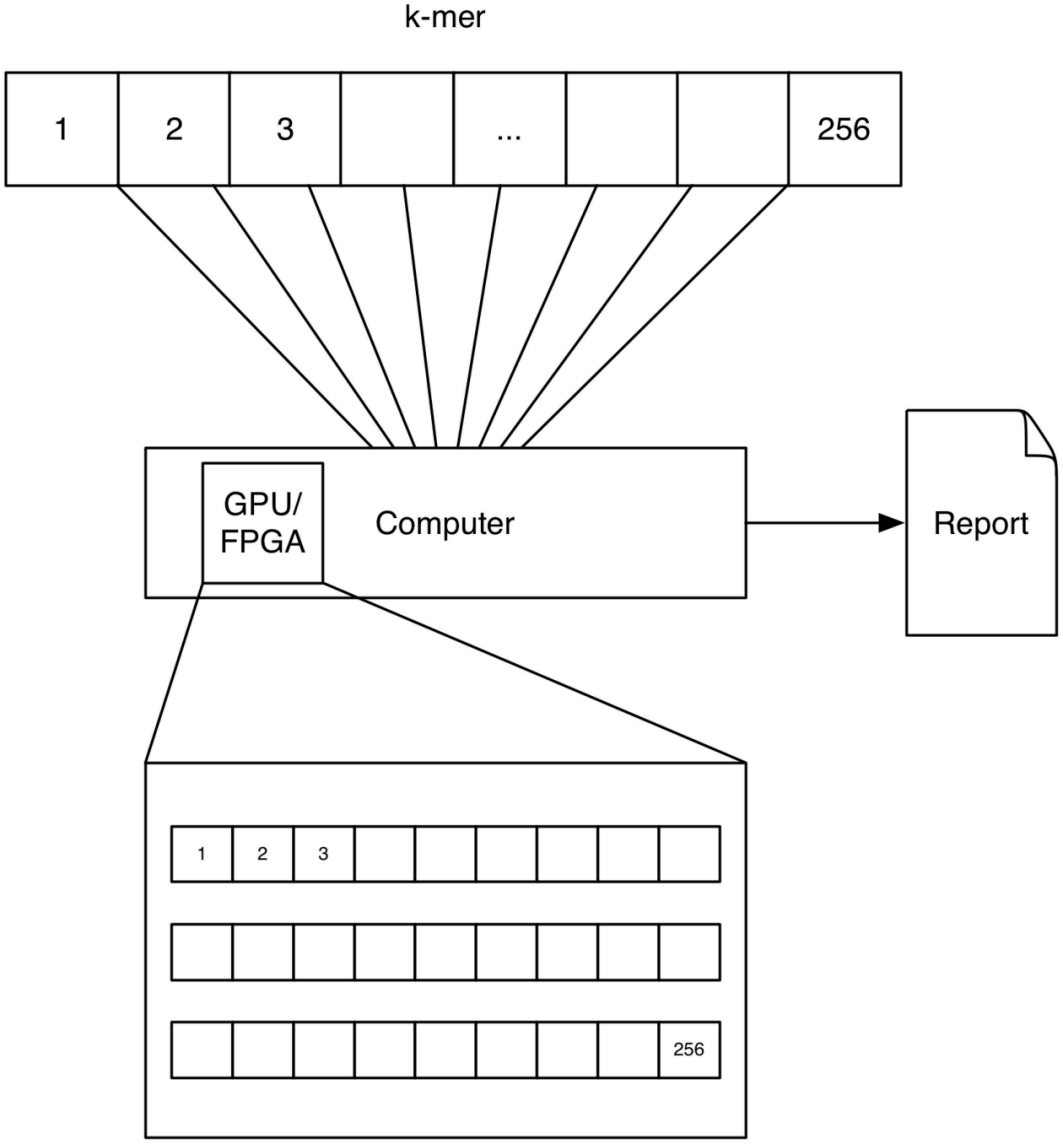
**GPU/FPGA: The concept of GPU and FPGA computing of ***k***-mers is similar, as both devices feature a multitude of processors**. Here the task is distributed to these processors by computing one *k*-mer on each processor. Note that for FPGAs a multitude of approaches exist for solving this problem and this solution is only represented as one possibility.

A schematic depicting one possibility of computing *k*-mers using GPUs is shown in Figure 3. Each processor on the GPU computes one *k*-mer count.

#### 2.1.4. FPGA

Field programmable gate arrays (FPGA) are configurable integrated circuits, unlike other integrated circuits in a computer that have a fixed layout. FPGA accelerator devices are a promising way to tackle several serious computation issues due to their hand tailored hardware solution with very fine-grained control over every aspect of execution. Unfortunately this is condition to an explicit and precise description of the algorithm and a cost intensive transformation of the algorithm to an FPGA design. In contrast to application development, the process of designing an FPGA circuit requires knowledge of a hardware description language as well as a general understanding of hardware design. Furthermore, some benefits of CPU programming as cache architectures or floating point numbers are not available, but can be implemented involving a lot of effort and extensive knowledge. In order to overcome the gap from software to hardware development, high-level synthesis tools such as the ROCC compiler (Villarreal et al., 2010), being able to transform C source code into an FPGA design, are available but very limited in terms of supported language constructs. In addition to the requirements for FPGA design, unit costs per FPGA are much higher when compared to multi-processor CPUs or GPU computing-capable graphics cards.

A schematic depicting one possibility of computing *k*-mers using FPGAs is shown in Figure 3. The FPGA is configured to have a counter for every *k*-mer. Each counter counts the occurrence of a *k*-mer at the current text-position. Please note that we describe a solution, which used the FPGA to solve the problem in a parallel way in order to speed it up. Further solutions exists which exploit the fine grained control of the configurable hardware, e.g., accelerating data throughput by setting the size of a character to 2 bit for a DNA alphabet.

#### 2.1.5. Cluster computing

##### 2.1.5.1. Message passing

A high performance computing setup with message passing consists of several computers interconnected by low-latency links and working in a parallel manner by partitioning a computational problem into sub-problems, which are solved on each computer. The computers require communication to efficiently share data, exchange intermediate results and thus gave birth to the message passing interface (MPI), implemented by MPICH (Bridges et al., 1995) as well as the more recent Open MPI (Gabriel et al., 2004). A problem is well suited for solving with MPI if interconnected computers require little communication between each other, as waiting for responses from other computers would significantly lower the performance. Careful thought has to be given to partition the problem into sub problems to limit communication. This requires an extensive knowledge about the algorithm. Furthermore, the source code has to be modified to solve the sub-problems and employ message passing. To the authors knowledge, there are only very few rudimental approaches like in Bondhugula (2013) available considering techniques to automatically port code for MPI execution.

**Figure 4:**
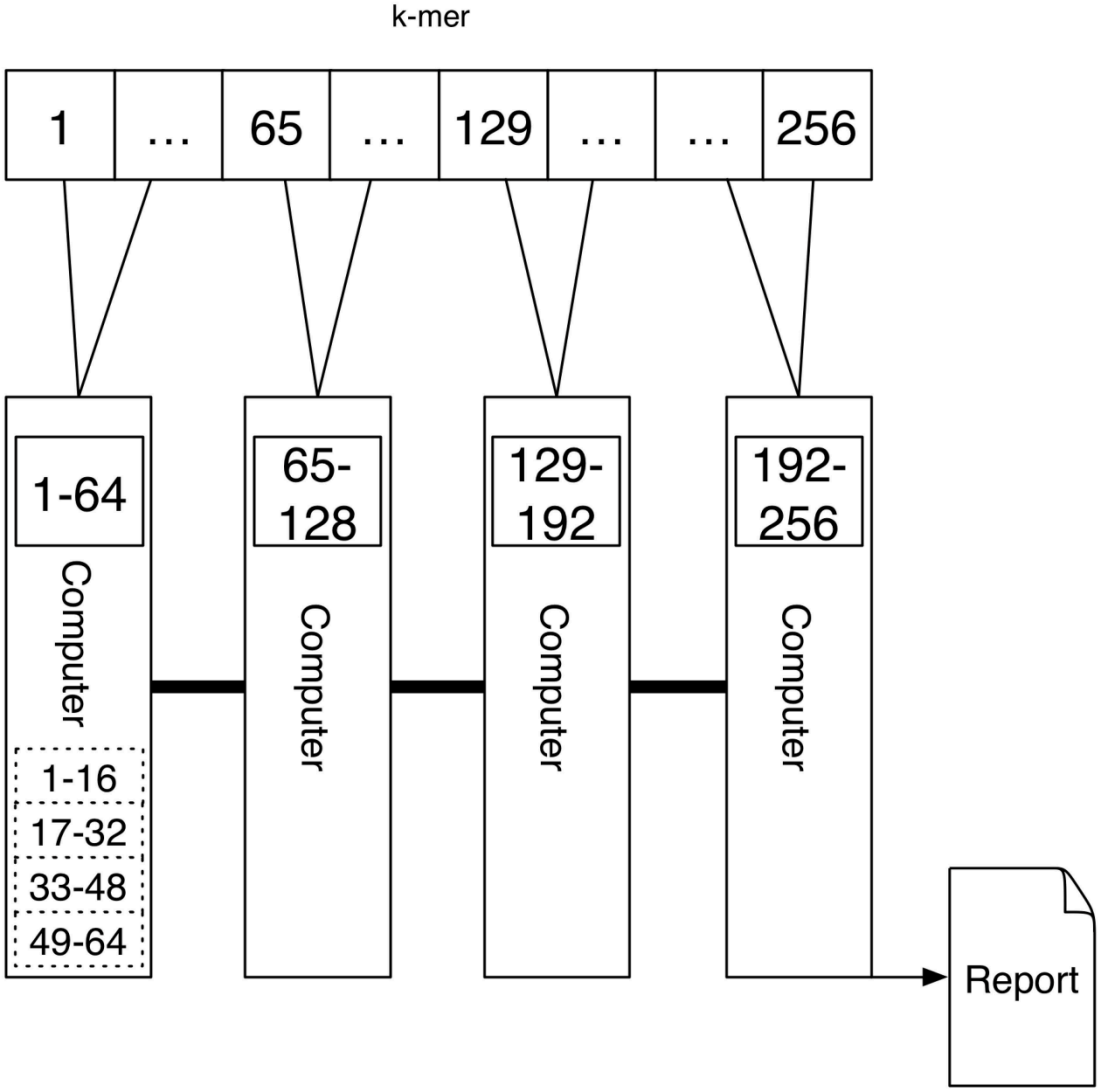
**An interconnected network of computers is given**. Each computer might have a single processor or multiple processors (depicted by the dashed rectangular boxes). The task is first distributed to the four computers, where a quarter of the *k*-mer counts are computed. If there are multiple processors the work is further distributed to these. Because the tasks are distributed to multiple computers the report cannot be produced directly since the results are present on different devices. Using the connection between the computers one device is gathering the results using Message Passing.

A schematic depicting one possibility of computing *k*-mers using Message Passing is shown in Figure 4. Here each computer computes the *k*-mer counts of a portion of all *k*-mers independently. The counts are transferred via Message Passing to one computer that generates the report.

##### 2.1.5.2. Job scheduling cluster computing

A compute cluster in the job scheduling setting is a loosely coupled set of autonomous computers that are connected to a central server acting as scheduler. The scheduler receives compute job requests and spawns them on computers in the cluster. Job scheduling cluster computing is feasible for most applications, as in general no cluster specific functionality has to be provided by the application. However, the data to be processed must be dividable into independent chunks that can be processed independently on different machines without any communication.

Unlike in message passing, computers are often either interconnected via a slow, regular network connection or not interconnected at all since communication between jobs is not intended in this setup. Different scheduling software solutions exist e.g., Oracle grid engine (Developers, 2013), the Univa Grid Engine™ (Univa Corporation, 2013) or Simple Linux Utility for Resource Management (Yoo et al., 2003) and a common mechanism for controlling jobs on compute clusters has been established with the distributed resource management application API (DRMAA; Rajic et al., 2004). But unfortunately the scheduler software has to be configured which is non-trivial.

**Figure 5:**
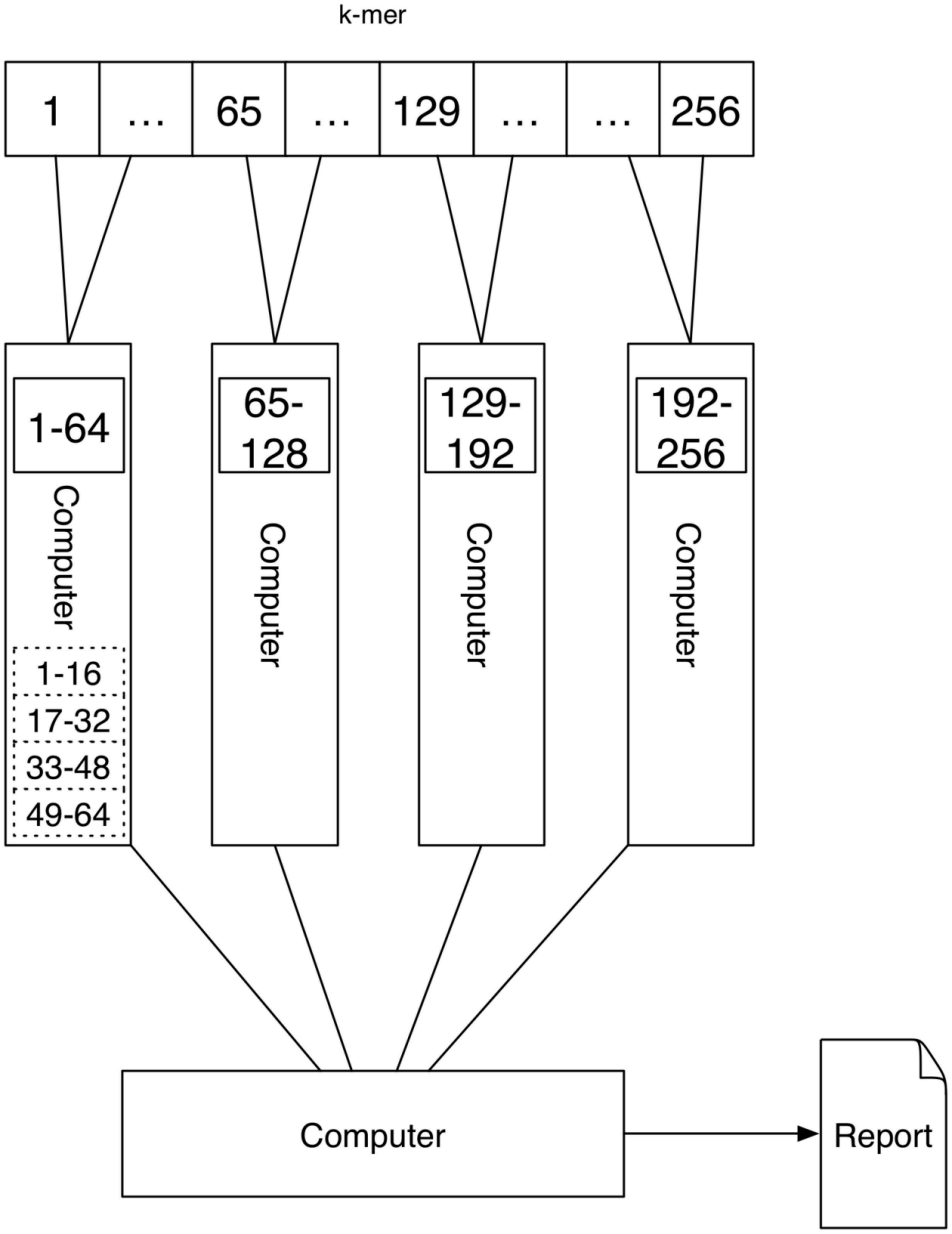
**Job scheduling cluster computing: The setup of Job scheduling cluster computing is similar to that of Message Passing**. There are four computers with a single or multiple processors that execute the task of counting the *k*-mers. However, there is no communication between the computers. Therefore, each computer stores its results to hard disk. After all computers have finished the *k*-mer counting the results have to be read from hard drive and are gathered to be able to output the report.

A schematic depicting one possibility of computing *k*-mers using Job scheduling cluster computing is shown in Figure 5. For each *k*-mer an application (counting one *k*-mer in one text) is executed, which receives the *k*-mer to be counted, as well as the file containing the sequence in which the *k*-mer should be counted as parameters. These are spawned on the computers of the cluster by the scheduler. Because all applications are independent of each other, an application needs to be started which waits for all jobs to finish and gathers the results by reading them from hard-drive.

##### 2.1.5.3. Further approaches

Another popular programming paradigm working on top of MPI (MapReduce-MPI) or the more popular Apache Hadoop is MapReduce (Dean and Ghemawat, 2008). The problem to be solved is described as a pair of a map and a reduce function. Each application of the map function to a data point is run as an independent process on multiple computers, while the reduce function aggregates the results. The advantage of MapReduce is that the programmer only needs to reformulate the problem at hand as map and reduce function and the parallelization and data migration are handled by the MapReduce framework. However, it must be possible to express the problem as a series of map/reduce functions. Furthermore, the computers must be all connected to each other and the speed of the reduce step might be dependent of the bandwidth/latency of the network.

A special kind of job scheduling cluster computing setting is grid computing. A grid is a “flexible, secure, coordinated resource sharing among dynamic collections of individuals, institutions, and resources” (Foster et al., 2001). Although a grid being a very abstract concept nowadays people describe the grid as more heterogeneous, loosely coupled and spatially diverse as a regular job scheduling cluster computing system.

### 2.2. Establishment of parallel processing in sequence informatics

The advent of novel sequencing technologies around 2005, namely the 454 GS by Roche (Margulies et al., 2005), the Illumina GAIIx (Bentley et al., 2008) and the Applied Biosystems SOLiD system (Shendure et al., 2005) marked a new era in nucleotide sequencing as well as bioinformatics. Genome sequencing projects that previously took months to finish now could be processed in several days. Thus, the amount of data generated significantly increased by several orders of magnitude, rendering many well-established bioinformatics applications of the Sanger-era obsolete.

Compared to Sanger sequencing (Sanger et al., 1977), which is able to process up to hundreds of samples in parallel on current machines, all NGS systems are able to sequence millions of DNA samples in parallel. Not only the pure amount of reads increased, but the length of the reads also decreased noticeably. Reads originating from traditional Sanger sequencing spanned more than 1000 bp, reads generated by the 454 system achieved lengths of 100 base pairs and reads acquired by Illumina machines were limited to 36 base pairs at best in the early days of next generation sequencing.

#### 2.2.1. Sequence comparison methods/algorithms

With the introduction of steadily evolving DNA sequencing techniques to decipher the order of nucleotides one obvious question is the measurement of similarity of two or more nucleotide sequences. As such, sequence comparison is one of the core methodologies in bioinformatics. It is used for various purposes such as database search (Pertsemlidis and Fondon, 2001), short read mapping (Li and Homer, 2010), multiple alignment of sequences (Thompson et al., 2011) with application in e.g., phylogenetics, assembly, proteomics and comparative genomics. Because of its ubiquitous usage for the field, various approaches for each of the domains exist and many of the applications incorporate parallelism. Furthermore, some applications exploiting algorithmic properties for fast computation exist which use e.g., *k*-mers instead of comparing string sequences, with some also using parallelization techniques (McHardy et al., 2007; Langenkämper et al., 2014). In database searches for instance, fast alignment algorithms are required that are capable to handle the increase in database sizes and the increase of queries to these databases. The most popular application for sequence database search is the Basic Local Alignment Search Tool (BLAST; Altschul et al., 1990).

Short read mapping requires algorithms that are capable of aligning a large number of sequences to a relatively small database (compared to typical BLAST databases) with few errors. In multiple sequence alignment, comparison algorithms are used for phylogenetic analyses. As such they require a high sensitivity given an evolutionary model and a number of related sequences.

#### 2.2.2. BLAST

Sequence database search in bioinformatics is tightly connected to BLAST. The core functionality of BLAST is to find sequence similarities on nucleotide and protein level; it is often run as part of genome annotation pipelines. The annotation process employed today would hardly be possible if applications used on a daily basis would be limited to one CPU on a single machine. BLAST can profit from execution on multi-processor computers, as it may use a specified number of CPUs on one computer or, on a larger scale, running on job scheduler systems, distributing the input data to multiple computers as well.

Additionally, adapted versions of BLAST running on special purpose hardware (FPGA: TimeLogic ®Tera-BLAST ™; TimeLogic Division, 2013; GPU: GPU-BLAST; Vouzis and Sahinidis, 2011, G-BLASTN; Zhao and Chu, 2014) exist.

#### 2.2.3. Read mapping

Different use cases for sequencing genomic DNA exist. An organism's genome can be sequenced for the first time, resulting in a *de novo* approach, or an organism closely related to a known organism is sequenced, hence a re-sequencing is carried out. In many cases different but closely related strains of a species are sequenced to obtain knowledge about genomic differences between two or more individuals. In this case all reads resulting from sequencing may be mapped against a known reference. This task, known as read mapping, is another topic that gained attention since the advent of NGS technologies. Typical representatives are MAQ (Li et al., 2008), SSAHA (Ning et al., 2001), BLAT (Kent, 2002), BLASTZ (Schwartz et al., 2003), GMAP (Wu and Watanabe, 2005), SOAP(-dp) (Li et al., 2008; Luo et al., 2013), Bowtie(2) (Langmead et al., 2009; Langmead and Salzberg, 2012), BWA (Li and Durbin, 2009, 2010), BarraCUDA (Klus et al., 2012), CUSHAW (Liu et al., 2012b), CUSHAW2-GPU (Liu and Schmidt, 2014). SOAP, BWA and Bowtie have been under active development since their release, resulting in an implementation for use on FPGA systems (Convey Computer, 2011). SOAP was released in a 2nd version (Li et al., 2009) and eventually ported to GPUs (Liu et al., 2012a; Luo et al., 2013) using a CUDA backend. More GPU compatible tools like BarraCUDA, CUSHAW(2) are available.

#### 2.2.4. Assembly

Nowadays, all available sequencing methods rely on fragmenting the input DNA. Therefore, it is crucial to be able to re-obtain the correct order of fragments after sequencing has taken place. This task is known as sequence genome assembly. Different algorithmic approaches have been proposed to solve this problem; two prominent examples are De Bruijn graph-based approaches (Myers et al., 2000; Pevzner et al., 2001; Batzoglou et al., 2002; Sommer et al., 2007; Butler et al., 2008; Miller et al., 2008; Zerbino and Birney, 2008; Simpson et al., 2009; Boisvert et al., 2010; Li et al., 2010) and overlap-layout-consensus (OLC) based (Dohm et al., 2007; Jeck et al., 2007; Warren et al., 2007; Bryant et al., 2009; Hossain et al., 2009; Ariyaratne and Sung, 2011) assembly applications. When confronted with data sets in human genome scale hardware requirements, specifically memory consumption, are increasing drastically for the assembly problem. Unfortunately, parallelization is not trivial for assembly algorithms, resulting in staged approaches where only several stages of the assembly process are distributed to multiple processors. Thus, assembly algorithms have only been ported to work with compute cluster infrastructures (Myers et al., 2000; Simpson et al., 2009).

State-of-the-art server systems possess up to 2TB of main memory, allowing even for *de novo* assemblies of organisms with very large and complex genomes. However, the maximal amount of memory per GPU is limited to 12 GB [NVIDIA Tesla K40 (NVIDIA Corporation, 2014), NVIDIA Tesla K80 2 × 12 GB (NVIDIA Corporation, 2015)]. Until now, to the authors' knowledge no GPU-based assembly software has been published. With a larger GPU memory in the future an adaptation of existing assembly algorithms for GPUs or the development of new approaches able to fully exploit all of their advantages may significantly lower the time required for large NGS assemblies. However, one major drawback of GPU-driven approaches often is the bottleneck for data transfer between host system and GPU.

## 3. Results

In the following, tools that automatically employ an optimization technique are evaluated. The techniques tested are CPU-Multi-processing, Vector instructions and cache optimizations, and GPU Computing. They are evaluated using two use cases. One is the counting of a pattern in a number of sequences, e.g., a *k*-mer as presented in the Introduction. The second task is the multiplication of two large matrices, which is an example more fitted for most optimizers. Furthermore, matrix multiplication is relevant as a building block for a multitude of sequence or general bioinformatics problems, e.g., Hidden Markov models or clustering. The C code presented in the following listings lists only the part that is computationally expensive for the given task. This part is also called the kernel or hotspot.

### 3.1. Settings

The examples were run on 2 x Intel Xeon E5620 (2.4 GHZ, 4 cores, hyperthreading enabled) CPUs with 70GB RAM and a NVIDIA Tesla C2070 GPU. The compilation was done using the following tools. Optimization flags were used if applicable. The source code is provided in the Supplementary Material.

For compilation the following tools were used:

- **C/C++** gcc 4.92
- **CUDA** nvcc 7.5
- **PPCG** ppcg 0.04 + nvcc 7.5
- **PluTo-SICA** PluTo 0.10.0-100-g45b91e4, current (11.11.2015) SICA Github branch + gcc 4.92
- **OpenACC** pgcc 15.7-0

### 3.2. Evaluation

**Figure 6:**
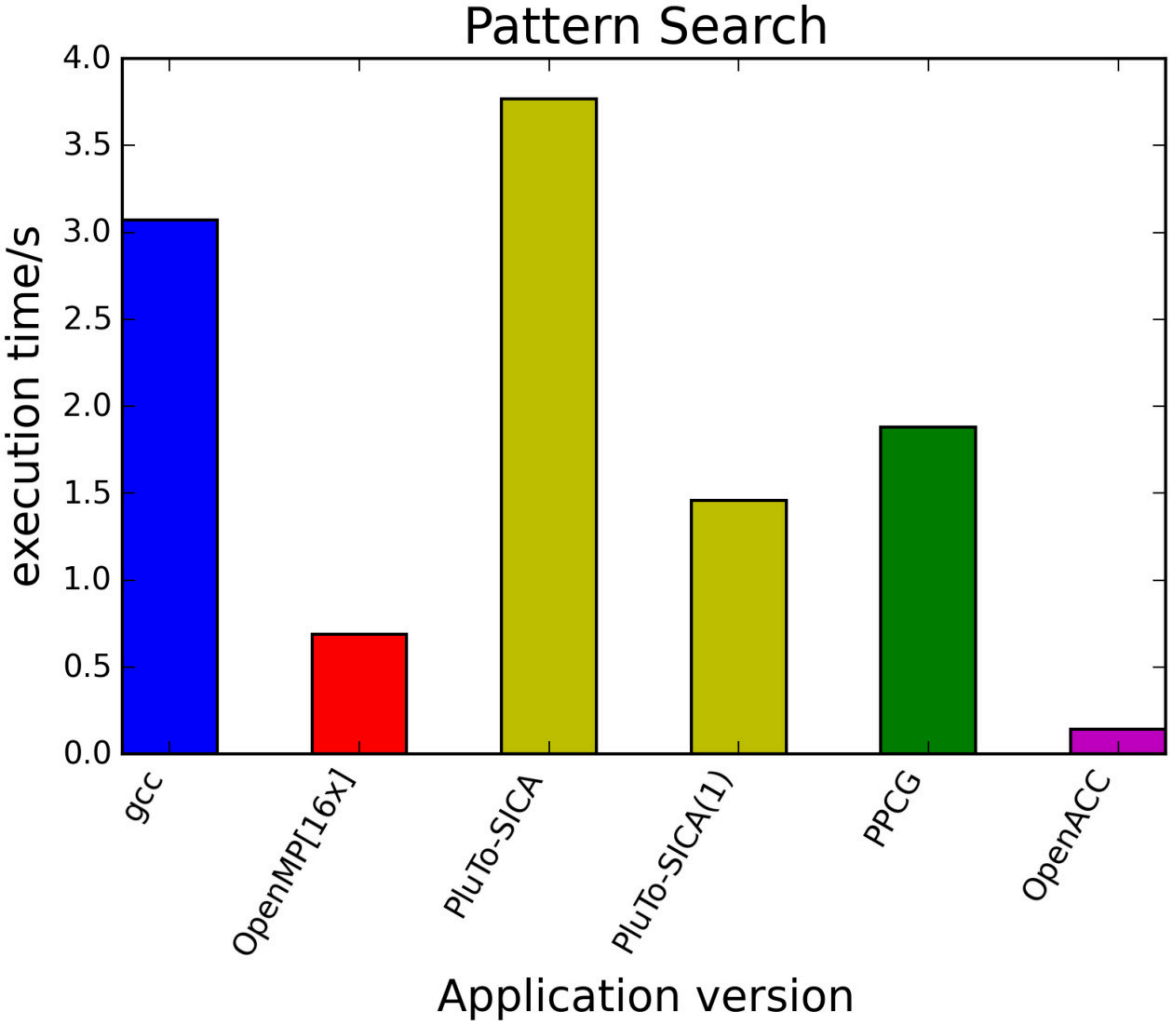
**Comparison of different automatic optimization techniques for pattern counting**. 200,000 sequences each with a length of 5000 base pairs are analyzed. PluTo-SICA(1) is a version with manual loop unrolling (see Supplementary Material, listing 12).

**Figure 7:**
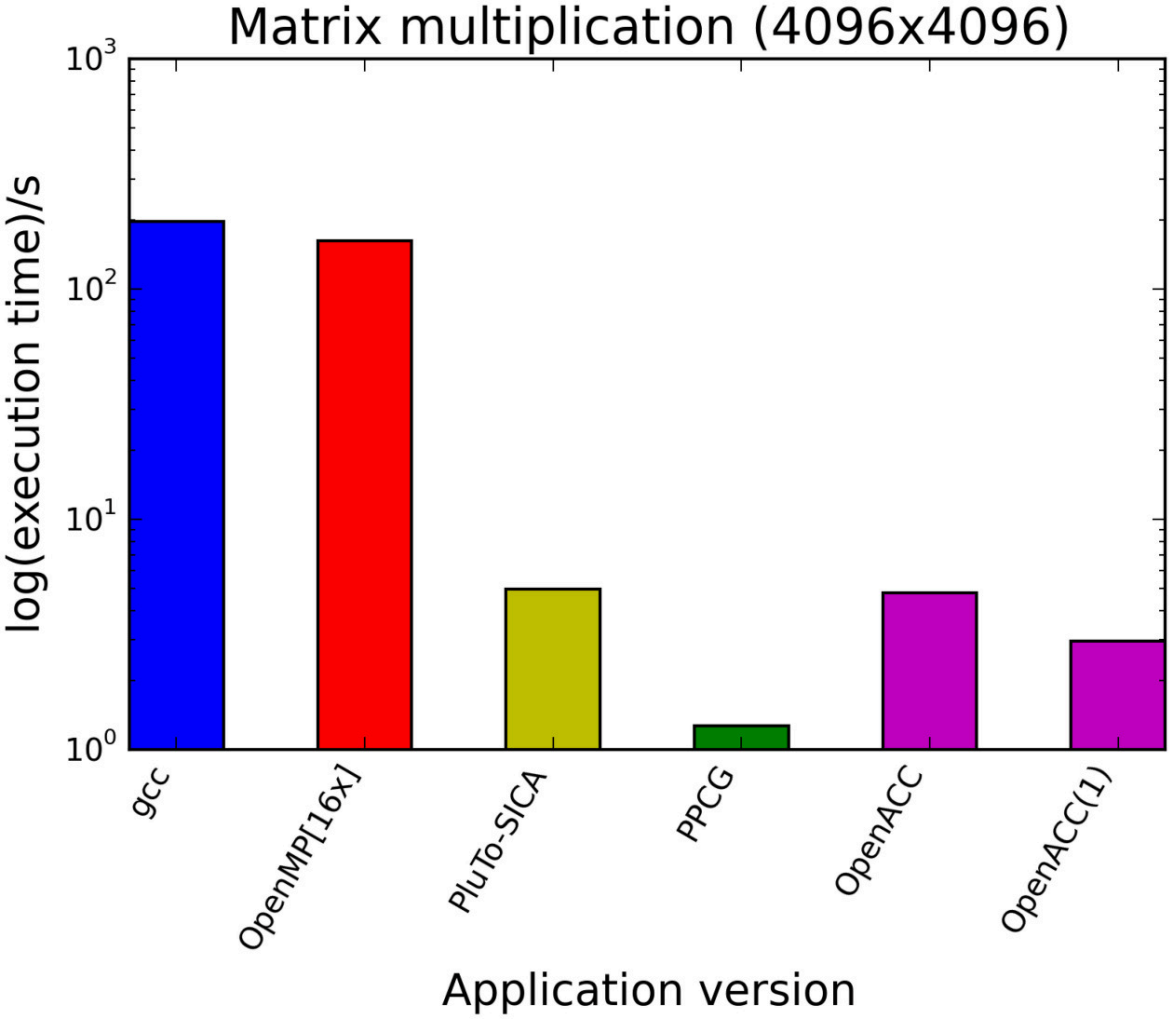
**Comparison of different automatic optimization techniques for matrix multiplication**. Two 4096 × 4096 matrices are multiplied. OpenACC corresponds to the time needed only for computation neglecting the time needed for driver initialization. OpenACC (1) is a version introducing a temporary variable (see Supplementary Material, listing 4).

**Figure 8:**
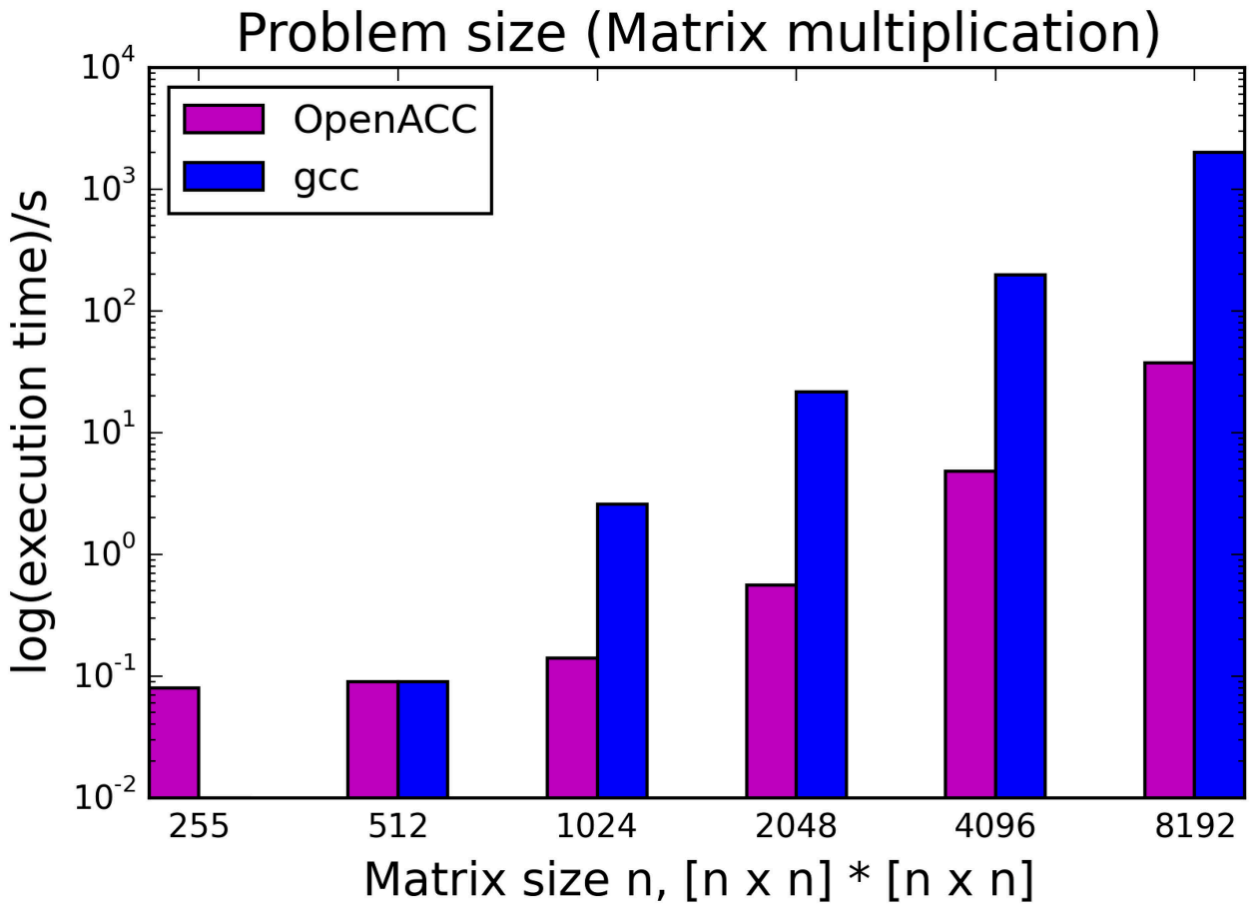
**Influence of problem size on the runtime of the serial version (gcc) compared to the OpenACC version**.

**Figure 9:**
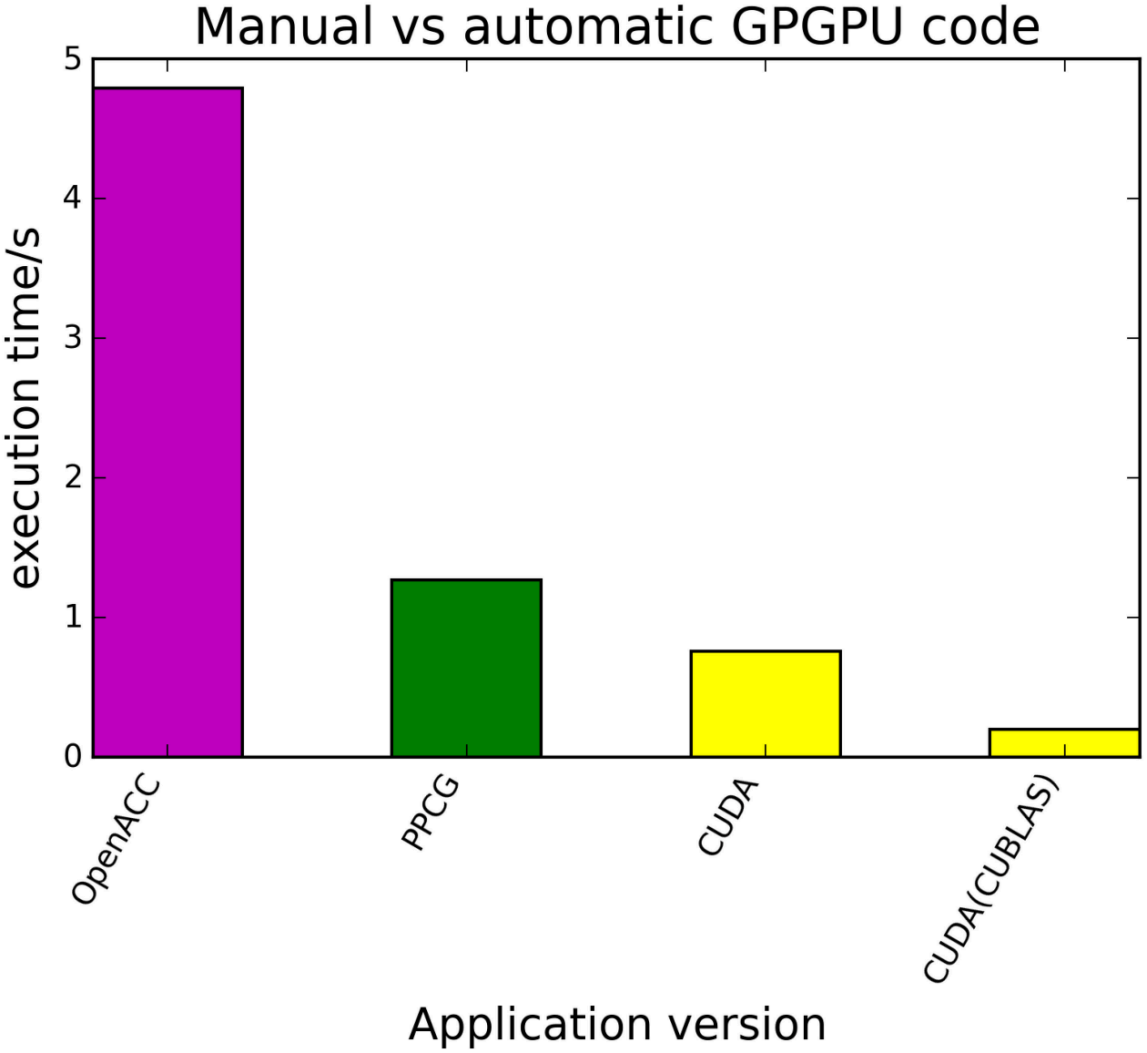
**Comparison of automatically transformed code (PPCG, OpenACC) and manually optimized CUDA Code for the matrix multiplication of two 4096 square matrices**.

The kernels' runtimes were measured and are reported in Figure 6 (Table 1) and Figure 7 (Table 2). It can be seen that significant speedups can be achieved using GPUs as show in the matrix multiplication example (see Figure 7; Table 2, PPCG/OpenACC). In the pattern counting example the speedup using GPUs is modest for PPCG but significant for OpenACC. Most optimization techniques introduce an overhead, which means that the problem size needs to be reasonably large to measure a speedup (see Figure 8; Table 3). Finally the comparison of manually optimized CUDA code written by developers of NVIDIA CUDA [see Figure 9; Table 4 CUDA/CUDA (CUBLAS)] compared to automatically optimized GPU Code generated by OpenACC (see Figure 9; Table 4 OpenACC) shows that the manually optimized code is up to one order of magnitude faster. For the CPU-based approaches [PluTo-SICA (multithreading and cache optimization) and OpenMP (multithreading)] the speedup varies with the provided task. For the matrix multiplication, PluTo-SICA outperforms OpenMP significantly (see Figure 7; Table 2 OpenMP/PluTo-SICA). This is due to PluTo-SICA's cache optimization. In the OpenMP version, the memory access patterns are not specifically optimized. PluTo-SICA divides the matrix multiplication into smaller blocks that fit in the cache to improve performance (Lam et al., 1991). When looking at the pattern counting example, OpenMP outperforms PluTo-SICA (see Figure 6; Table 1) and, even more, the PluTo-SICA version is slower than the original code. This is caused by the skewing of different loops in PluTo-SICA's tiling step (tiling/blocking is the process of reordering the loop iteration sequence to reuse local data better), including the short innermost k-loop, which iterates only four times per loop. This tiling transformation blocks the code for improved data reuse on the one hand but, on the other, it makes the resulting code way more complex than the original version and therefore restricts the compiler's (in our case “gcc”) capability of optimizing the resulting source code furthermore. Additionally, the blocking for better cache usage does not pay out for this example code as the additionally obtained reuse of data is, due to the code's properties, relatively small. For the original code, a compiler will in general be able to unroll the aforementioned inner short loop completely and replace it by four instructions or even vectorize it directly. After PluTo-SICA's transformation, this is not the case anymore due to the complex resulting loop structures caused by the new iteration order.

**Table 1:** **Runtimes of pattern counting**.

**Application version**	**Runtime (s)**
gcc	3.07
OpenMP [16 threads]	0.69
PluTo-SICA	3.77
PluTo-SICA manual loop unroll	1.46
PPCG	1.88
OpenACC	0.14

*200,000 sequences each with a length of 5000 base pairs are analyzed*.

**Table 2:** **Runtimes of matrix multiplication of two 4096 square matrices**.

**Application version**	**Runtime (s)**
gcc	196.99
OpenMP [16 threads]	161.8
PluTo-SICA	4.97
PPCG	1.27
OpenACC	4.79
OpenACC w tmp	2.95

**Table 3:** **Runtimes of matrix multiplication with different problem sizes**.

***n***	**OpenACC runtime**	**gcc runtime**
256	0.08	0.01
512	0.09	0.09
1024	0.14	2.56
2048	0.56	21.38
4096	4.79	196.99
8192	37.05	1982.70

**Table 4:** **Runtimes of automatically transformed code vs. manually optimized code for the matrix multiplication of two 4096 square matrices**.

**Application version**	**Runtime (s)**
OpenACC	4.79
PPCG	1.27
CUDA (matrixMul)	0.76
CUDA (matrixMulCUBLAS)	0.2

## 4. Discussion

Throughout this study, a broad spectrum of different parallelization strategies was presented and a subset evaluated. Our results show that the performance improvement can be quiet formidable, as shown by PPCG optimized matrix multiplication code that achieves a **155 ×** speedup compared to the runtime of the source code optimized by gcc. The comparison of GPU-ported applications with multi-processor applications of the same algorithm shows in most cases a significant speedup favoring the GPU implementation. However, this speedup is only applicable if the problem size surpasses a certain threshold, meaning that for small input sizes an overhead added by data migration and driver initialization reduces the performance (Figure 8; Table 3). The effect of this overhead on the total runtime is shown by OpenACC in Figure 7; Table 2. Unfortunately the estimation of the problem size beyond which a port of an application to GPUs is reasonable, is difficult. Automatic transformation tools lower the resources required to transform an application such that these boundaries may be determined empirically. Our evaluation shows that while the OpenMP-Code performs better on the task of counting a pattern in a set of sequences (cp. chapter *Results - Evaluation* for an explanation), PluTo-SICA's optimization outperforms the OpenMP code in the case of the matrix multiplication by one order of magnitude. While PPCG performs better in the matrix multiplication example, OpenACC performs significantly better in the pattern counting example. This is due to different approaches for optimization. Though results may appear impressive, tools for automatic transformation of source code are limited. This may be owed to technical difficulties or to the lack of maturity of the compiler. It should also be noted that some optimizers are still in the development phase during which additional language features and constructs may be added in the future.

OpenMP is one of the most mature parallelization techniques employed, and well integrated into several compilers. It imposes almost no limitations. OpenACC compiler support is limited to few commercially available products while open source alternatives such as in gcc are still in an early stage of development. The novelty of these compilers and the complex nature of memory layout and execution on GPUs impose limitations in the automatic transformation of arbitrary code. PPCG and PluTo-SICA employ a polyhedral model to optimize applications, which is a well-known and established tool especially in academic research. But there are restrictions concerning the source code, which can be transformed efficiently. Furthermore, these are novel applications, which are relatively unestablished.

Much progress has been made in the bioinformatics domain in recent years to introduce parallelization techniques. Most serial applications feature multi-processor implementations or have been ported to GPU or FPGA hardware. Assembly software, read mapper and other “-omics” related software is at least partially parallelized. In contrast to other domains of informatics related to natural sciences that tend to employ floating point calculations, the majority of bioinformatics software is partially string-based due to the relation to DNA or protein sequences. This kinship includes data transfer and storage of large sequences that currently hardly fit into available GPU memories or even FPGA circuits.

CPU Multi-Processing and Vector Instructions are established in the bioinformatics community and hardware is ubiquitous. OpenMP is a non-invasive easy to apply approach. PluTo-SICA is not that established but seems to be a promising tool in the future. Furthermore, regular compiler software such as gcc, icc, or llvm also employ cache optimization and automatic transformation to vector instructions, but the outcome is often less satisfactory.

Projecting the current status of GPU computing, it is reasonable to assume that in a few generations GPU memory capacity may be large enough to fit whole data sets and allow for complete applications to be ported to GPUs rather than just to subsets of the application with the downside of data migration. This would minimize the required data transfer and theoretically allow for BLAST-like implementations able to store whole BLAST databases into the GPU memory. OpenACC, although only commercially available, is a rather mature and efficient tool. PPCG lacks support and is limited, but it is free to use and our results are comparable to OpenACC. Given the current pace of development of optimization techniques, it seems reasonable that in several years tool chains will be able to automatically parallelize most language constructs with less restrictions.

FPGAs are specialized hardware, which is expensive and hard to use. Because manually drafting an FPGA design is so different compared to regular software design, the effort is often not worth it for a software engineer. Except a few sold applications (so called personalities), FPGA applications are hardly present in the bioinformatics community. On the other hand FPGAs can be a promising possibility for problems, which are otherwise not solvable under certain constraints. The automatic optimizer ROCC compiler is immature and rather limited in the code it can transform. Please note that techniques for utilizing the full potential of an FPGA as well as an extensive review of FPGA optimization techniques is out of the scope of this paper.

Job scheduling cluster computing is easy to apply if the problem is fitted. If the application usually processes multiple input files independently one can start one application for each input file on a cluster computer instead. If the input files are not independently processed or intermediate results have to be computed or gathered, job scheduling cluster computing is rather slow because then data has to be read and written to and from hard disk via network. Furthermore, the maintenance of a (large) cluster is non-trivial.

Message Passing programming is nowadays mostly used on supercomputers and large-scale computer networks. The hardware used is expensive and hardly any automatic transformation tools exist. On the other hand it is the method of choice to use the compute power of more than one computer efficiently (for a problem not fitted for job scheduling cluster computing). In modern MPI applications a problem is partitioned into sub-problems which are distributed using MPI and are parallelly solved using GPUs or CPU-Multi-Processing.

The simultaneous utilization of multiple acceleration techniques is possible, though not all combinations of techniques are easy to implement (see Supplementary Figure 1). CPU based techniques (CPU-multiprocessing, vector instructions and cache optimization) are straightforward combinable and most compilers implement all these optimizations. Combining cluster based approaches (Job scheduling, MPI) with CPU based approaches or accelerator based approaches (GPU, FPGA) is straightforward as well, as mentioned above. Utilizing CPU and accelerators simultaneously is not that easy. Dynamic effective load balancing and check-pointing are key to peak performance and full utilization of both systems. Although some successful implementations exist, memory migration and dramatic differences in performance often lead to an inefficient utilization. Furthermore, automatic approaches for CPU-Accelerator co-execution do not exist to the authors' knowledge.

**Figure 10:**
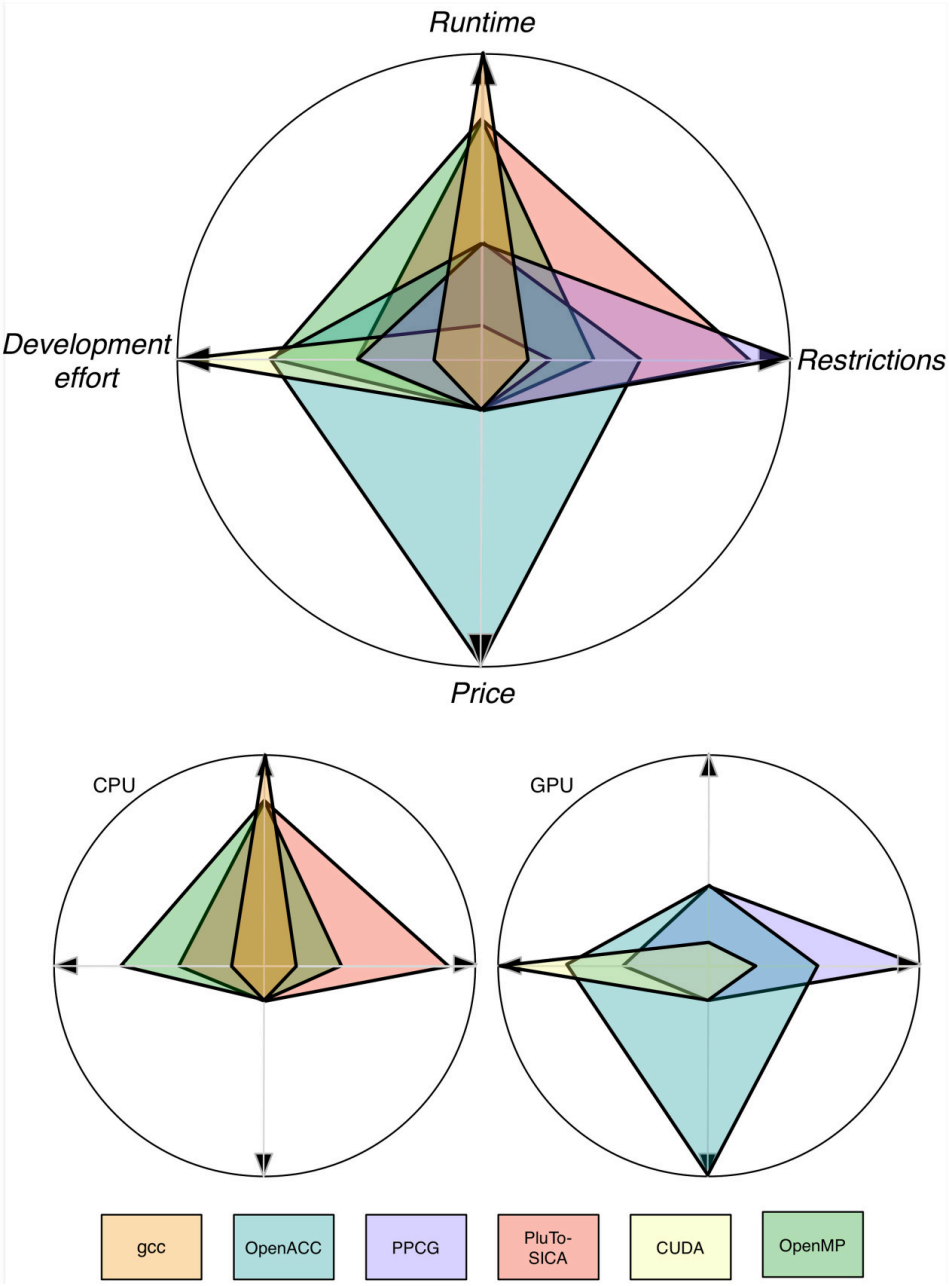
**Trends for four categories depicting the pros and cons of different optimization techniques**. Note that these values are only trends and the depicted values are problem dependent. Note that the runtime axis is scaled logarithmically.

As shown throughout this study, automatic parallelization is possible and has shown its feasibility (Figure 10). The easiest approach seems to be to use CPU-Multi-Processing using OpenMP and vector instructions/cache optimization because no special hardware is needed, automatic transformation is easy to use and it is applicable to a brought range of source code. If a major speed up is desired and the respective hardware is available as well as the code is suitable, GPU application using automatic transformation with OpenACC is advisable as the speed-up is reasonable for the effort to learn to apply it. Unfortunately, only commercial products are available. FPGAs, compute clusters and MPI are special interest products, which are expensive and/or hard to use. Unfortunately most bioinformatics software does not yet profit from these techniques. But it is reasonable to think that in the future improvements in hardware and advances in the automatic optimization tools will make it easier for the bioinformatics community to profit from parallelization.

## Author contributions

DL, TJ, LJ, and TN designed the work while DL, TJ, DF, AG, and TN interpreted the findings. DL and DF implemented the routines. DL, TJ, DF, and LJ drafted the paper and AG and TN revised it carefully. DL, TJ, DF, LJ, AG, and TN gave their final approval of the current version. DL, TJ, DF, LJ, AG, and TN gave their agreement to be accountable for all aspects of the work.

### Conflict of interest statement

The authors declare that the research was conducted in the absence of any commercial or financial relationships that could be construed as a potential conflict of interest.
