# Supplementary material for: Comparison of Acceleration Techniques for Selected Low-Level Bioinformatics Operations
Source: Front Genet. 2016 Feb 10;7:5. doi: 10.3389/fgene.2016.00005 (PMC4748744; doi:10.3389/fgene.2016.00005)
Supplement: Supplementary file 1 [file Supplementary.PDF]

Supplementary Material: State-of-the-art parallelization techniques for the bioinformatics community

Daniel Langenkämper [1,4], Tobias Jakobi [1,2,3], Dustin Feld [6], Lukas Jelonek [1,2,3,4], Alexander Goesmann[5] and Tim W. Nattkemper [1,4*]
*Correspondence:
Tim W. Nattkemper
tim.nattkemper@uni-bielefeld.de

1 SUPPLEMENTARY DATA

Matrix multiplication

```
for (i=0;i<M;i++){
    for(j=0;j<N;j++){
        for(k=0;k<K;k++){
            C[i][j]+=A[i][k]*B[k][j];
        }
    }
}
```

Listing 1. C: The plain C code takes two matrices A and B with $A \in \mathbb{R}^{M \times K}$ and $B \in \mathbb{R}^{K \times N}$, multiplies them and stores the results in $C \in \mathbb{R}^{M \times N}$.

```
 #pragma omp parallel for private(i,j,k)
for (i=0;i<M;i++){
    for(j=0;j<N;j++){
        for(k=0;k<K;k++){
            C[i][j]+=A[i][k]*B[k][j];
        }
    }
}
```

Listing 2. OpenMP: Parallelization with OpenMP uses a declarative approach. The highlighted declaration is added and is only considered if OpenMP is activated in the compiler. A “pragma omp parallel for” tells the compiler/OpenMP framework to parallelize the following loop. The “private(i,j,k)” term tells the compiler that every thread should have a private copy of these variables instead of one shared one.

```
 #pragma acc parallel loop pcopyin(A[0:SIZE],B[0:SIZE]) pcopyout(C[0:
   SIZE])
for (i=0;i<M;i++){
    for(j=0;j<N;j++){
        for(k=0;k<K;k++){
            C[i][j]+=A[i][k]*B[k][j];
        }
    }
```

```
}
```

Listing 3. .]**OpenACC:** Parallelization with OpenACC, like OpenMP, uses a declarative approach. #pragma acc parallel loop tells the OpenACC compiler to parallelize the following loop. pcopyin() checks if the data given as argument is already present on the accelerator. If it is not it copies it to the accelerator. pcopyout() checks if the data is present on the accelerator, otherwise it will be allocated and copied back from the accelerator to the host system after finishing the loop. The size of the data to be copied is given as variable[start:end].

```
 #pragma acc parallel loop pcopyin(A[0:SIZESQ],B[0:SIZESQ]) pcopyout(
   C[0:SIZESQ])
for (i=0;i<M;i++){
    for(j=0;j<N;j++){
        float tmp=0.f;
        for(k=0;k<K;k++){
            tmp+=A[i][k]*B[k][j];
        }
        C[i][j]=tmp;
    }
}
```

Listing 4. OpenACC with temporary variable: With the introduction of a temporary variable the optimizer tends to better parallelize the code because the static analysis of the code is easier for the compiler to do. Because there are no read/write accesses to C inside the inner loop the code can be further optimized (cmp. listing 5,6).

```
Complex loop carried dependence of C prevents parallelization
Loop carried dependence of C prevents parallelization
Loop carried backward dependence of C prevents vectorization
```

Listing 5. ppcg compiler output log for inner loop.

```
Loop is parallelizable
```

Listing 6. ppcg compiler output log for inner loop using a temporary variable.

```
 #pragma scop
for (i=0;i<M;i++){
    for(j=0;j<N;j++){
        for(k=0;k<K;k++){
            C[i][j]+=A[i][k]*B[k][j];
        }
    }
}
 #pragma endscop
```

Listing 7. PluTo-SICA/PPCG: Because PPCG and SICA share some underlying software the declaration is the same for both. The directives for optimization are also declarative but the code is not directly provided to a compiler but instead restructured by a respective transformation. Then the produced source code is passed to the compiler (gcc for CPU and nvcc for GPU). The #pragma scop and #pragmam endscop directives tell the optimizer which part should be accelerated.

pattern counting

```
for (i=0;i<numSeqs;i++){
    counter[i]=0;
    for(j=0;j<seqLength;j++){
        for (k=0;k<numPatterns;k++){
            counter[i]+=(seqs[i][j]==pattern[k]);
        }
    }
}
```

Listing 8. C The plain C code iterates through 200.000 sequences each with a length of 5.000 base pairs and counts the occurrences of a k-mer and stores the number in the array $counter$.

```
 #pragma omp parallel for private(i,j,k)
for (i=0;i<numSeqs;i++){
    counter[i]=0;
    for(j=0;j<seqLength;j++){
        for (k=0;k<numPatterns;k++){
            counter[i]+=(seqs[i][j]==pattern[k]);
        }
    }
}
```

Listing 9. OpenMP: Parallelization with OpenMP uses a declarative approach. The highlighted declaration is added and is only considered if OpenMP is activated in the compiler.

```
#pragma acc kernels pcopyin(seqs,pattern) pcopyout(counter)
{
#pragma acc loop independent
for (i=0;i<numSeqs;i++){
        counter[i]=0;
         #pragma acc loop seq
        for(j=0;j<seqLength;j++){
                 #pragma acc loop seq
                for (k=0;k<4;k++){
                        counter[i]+=(seqs[i][j]==pattern[k]);
                }
        }
}
}
```

Listing 10. OpenACC: Parallelization with OpenACC, like OpenMP, uses a declarative approach. In this case the kernels construct was used. The code included in this region is ported to the accelerator by the compiler. The #pragma acc loop independent directive tells the compiler that each iteration of this loop is independent from each other. In contrast to the #pragma acc loop seq directive which tells the compiler that there dependencies.

```
 #pragma scop
for (i=0;i<numSeqs;i++){
    counter[i]=0;
    for(j=0;j<seqLength;j++){
```

```
        for (k=0;k<numPatterns;k++){
            counter[i]+=(seqs[i][j]==pattern[k]);
        }
    }
}
 #pragma endscop
```

Listing 11. PluTo-SICA/PPCG: Because PPCG and SICA share some underlying software the declaration is the same for both. The directives for optimization are also declarative but the code is not directly provided to a compiler but instead restructured by a respective transformation. Then the produced source code is passed to the compiler (gcc for CPU and nvcc for GPU).

```
 #pragma scop
for (i=0;i<numSeqs;i++){
    counter[i]=0;
    for(j=0;j<seqLength;j++){
                counter[i]+=seqs[i][j]==pattern[0];
                counter[i]+=seqs[i][j]==pattern[1];
                counter[i]+=seqs[i][j]==pattern[2];
                counter[i]+=seqs[i][j]==pattern[3];
    }
}
 #pragma endscop
```

Listing 12. PluTo-SICA/PPCG: Pluto-Sica with manual unrolling of the inner loop. Note that this example is only true for pattern of length 4. We unrolled the short loop manually and measured the effect on the original source code and on the code generated by PluTo-SICA. The results showed that the manual unrolling has, as expected, no considerable effect on the performance of the original code but it has on PluTo-SICA's generated code (see PluTo-SICA(1) in Figure 6 (Table 1)). This has two main reasons, 1) the short loop in the resulting code is now unrolled and 2) the resulting loop structure after PluTo-SICA was applied is way simpler with unrolling by excluding the short inner loop from the transformation step in this way.

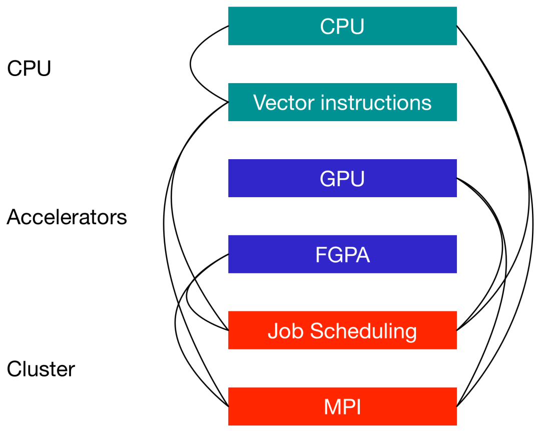

Figure 1. Favorable hybrid approaches.
